# How Not to Do a Mindset Intervention: Learning from a Mindset Intervention among Students with Good Grades

**DOI:** 10.3389/fpsyg.2017.00311

**Published:** 2017-03-09

**Authors:** Gábor Orosz, Szilvia Péter-Szarka, Beáta Bőthe, István Tóth-Király, Rony Berger

**Affiliations:** ^1^Faculty of Education and Psychology, Institute of Psychology, Eötvös Loránd UniversityBudapest, Hungary; ^2^Institute of Cognitive Neuroscience and Psychology, Research Centre for Natural Sciences, Hungarian Academy of SciencesBudapest, Hungary; ^3^Institute of Psychology, University of DebrecenDebrecen, Hungary; ^4^Doctoral School of Psychology, Eötvös Loránd UniversityBudapest, Hungary; ^5^Department of Emergency Medicine, Ben Gurion University of the NegevTel Aviv, Israel

**Keywords:** good grades, grit, growth mindset, incremental theory of intelligence, social psychological intervention

## Abstract

The present study examined the effectiveness of a Growth Mindset intervention based on Dweck et al.'s (1995) theory in the Hungarian educational context. A cluster randomized controlled trial classroom experiment was carried out within the framework of a train-the-trainer intervention among 55 Hungarian 10th grade students with high Grade Point Average (GPA). The results suggest that students' IQ and personality mindset beliefs were more incremental in the intervention group than in the control group 3 weeks after the intervention. Furthermore, compared to both the baseline measure and the control group, students' amotivation decreased. However, no intrinsic and extrinsic motivation change was found. Students with low grit scores reported lower amotivation following the intervention. However, in the second follow-up measurement—the end of the semester—all positive changes disappeared; and students' GPA did not change compared to the previous semester. These results show that mindset beliefs are temporarily malleable and in given circumstances, they can change back to their pre-intervention state. The potential explanation is discussed in the light of previous mindset intervention studies and recent findings on wise social psychological interventions.

## Introduction

The Incremental theory of intelligence—Growth Mindset since the book of Dweck (2006)—deals with beliefs influencing responses to challenges and setbacks (Dweck and Leggett, 1988; Hong et al., 1999). These beliefs refer to theories of students concerning the nature of intelligence. Students may believe that intelligence cannot be changed and it can be represented as a limited, stable entity (entity theory). Alternatively, they may believe that it can be developed (incremental theory) and further improved (for meta-analysis see Burnette et al., 2013). According to previous results, such beliefs have serious implications in terms of reactions to challenges, motivations, and choosing or giving up demanding activities (Dweck and Leggett, 1988; Mueller and Dweck, 1998). From the perspective of the societal significance of this theory, growth mindset can reduce the negative effect of poverty on academic achievement (Claro et al., 2016). From the perspective of cognitive neuroscience, growth mindset induction contributes to better cognitive control (Schroder et al., 2014). In the present study, we carried out a Mindset intervention which aimed to create incremental beliefs of intelligence among Hungarian high school students.

Based on Dweck's Mindset theory, several intervention studies were conducted with positive and promising results, mainly among underprivileged or minority groups. In one of the first interventions testing this theory, African-American students were encouraged to perceive their intelligence as a malleable rather than a fixed capacity (Aronson et al., 2002). This intervention made the performance of these students—compared to the control group—less vulnerable to stereotype threat, helped them in maintaining their academic engagement and also led to higher GPA. In a further study, Good et al. (2003) found that African-American, Hispanic and low income students' stereotype threat was reduced as a consequence of mentoring college students. These mentors encouraged the participants to see their intelligence malleable and helped them in attributing their academic problems to the novelty of educational context they were in. In another intervention among lower achiever students, focusing on the creation of incremental beliefs of intelligence had positive impact on classroom motivation, and it stopped the decline in mathematics grades compared the control group (Blackwell et al., 2007). More recently, Paunesku et al. (2015) carried out a large scale online field experiment in which they measured the effectiveness of a short (45 min) Mindset training. They found that exposing the incremental theory of intelligence raised the GPA among high school students who were at risk of dropping out. Consequently, these above-mentioned studies showed that the mindset intervention was mainly effective among those students who are African-American, Hispanic, lower achiever, who had lower income or who are at risk of drop-out.

The question arises whether Mindset trainings can be effective in a fundamentally different cultural and educational context: the Hungarian educational system (Csapó, 2015). In Hungary, efforts in general do not have a good reputation. For example, according to the fifth wave of the World Value Survey, only 7% of the Hungarians agreed that hard work pays off in the long run which is much lower than the total average of 19% (WVS, 2009). Furthermore, dissimilarly to previously measured high schools in the United States, the Hungarian public education can be characterized by rather conservative values, stronger state-level control of the national curriculum, and less supportive climate concerning change. In the present study, we aimed to test the effectiveness of growth mindset interventions in a fundamentally different school context as the US one. Furthermore, besides the potential less supportive educational context, we aimed to examine whether growth mindset can be effective among students who have relatively good grades.

In the present study, a Growth Mindset intervention was carried out for high-school students and its effect on academic motivations and grades was measured. The intervention protocol was similar to Aronson et al. (2002), Good et al. (2003), Blackwell et al. (2007) and Paunesku et al. (2015) and it was based on the precise and well-founded theory of the incremental theory of intelligence (Hong et al., 1999). Similarly to Blackwell et al.'s (2007), we intended to create a brief, offline intervention in which five classes (45 min) were spent with integrating the knowledge of incremental intelligence. Similarly to this study, it was a train-the-trainer intervention where the authors trained the teachers. We were mainly interested in the dynamics of the mindset beliefs and their short vs. long-term effects on the motivations of among students with good grades and the impact of intervention on their GPA. We expected that—as a result of the Growth Mindset intervention—students will be more motivated (H1) and will have higher GPA (H2).

Growth mindset beliefs are general beliefs that have a pervasive effect on different fields (see the meta-analysis of Burnette et al., 2013). Furthermore, previous interventions showed that there is a way to change them (Blackwell et al., 2007; Paunesku et al., 2015; Yeager et al., 2016). However, as far as we know, we have less accumulated knowledge about more stable individual differences—as personality traits—that might influence the effectiveness of these beliefs. One of the potential, proximal and stable individual differences or personality characteristics is Grit which refers to the perseverant and passionate striving toward long-term goals (Duckworth et al., 2007). Previous studies suggested that this characteristic contributes to academic success and success in many fields and in different age-groups (Duckworth et al., 2007; Duckworth and Quinn, 2009). We expected that the Growth Mindset intervention will be more effective among low Grit students compared to high Grit students (Duckworth and Quinn, 2009). We supposed that a Growth Mindset intervention—focusing on the importance of efforts and good strategies—can be less effective in terms of changing motivations and grades among those who are persistent and already make a lot of effort and strive for long-term goals compared to low Grit students whose motivations and grades can be improved by enhancing the importance of efforts (H3).

## Materials and methods

### Participants

A total of 123 Hungarian public high school students and their parents were told that they had the opportunity to participate in a 5-week workshop. In the case of the control group, they were informed that they could participate in an intervention related to helping behavior. In the experimental group, they were informed about the possibility to participate in a workshop about intelligence and learning.

Five parents and six students indicated that they did not wish to participate in the training. Therefore, 112 students participated in the pre-test, 79 students participated in the post-test and 55 students participated in the second post-test in this study in spring 2015 (from February to June). Causes of drop-out were non-participation in each round of the intervention (49 students), same code-names for different students (2 students), or code-names could not be matched with the students' real identity (6 students). These students were recruited from two Hungarian high schools from the countryside. Their average GPA right before the intervention was 3.72 (*SD* = 0.94) based on a 1 (unsatisfactory) to 5 (excellent) grading system. Students were 10th graders aged between 15 and 18 years (*M*_age_ = 16.00; *SD*_age_ = 0.58). A total of 26 students (11 female, 42.31%) were included in the intervention group and a total of 29 students (18 female, 62.07%) were in the control group.

To ensure the ethical treatment of human participants, this study was carried out with the approval of the local university's ethical board and it was conducted in accordance with the Declaration of Helsinki. Participation was entirely voluntary, and the consent of both students and parents were obtained in advance. Furthermore, the directors of the schools also approved the intervention in advance.

### Measures

All measures used in this study were translated to Hungarian from the original scales using the protocol of Beaton et al. (2000).

#### Intelligence mindset scale

We used the Implicit Theory of Intelligence Scale (Dweck et al., 1995) to assess the participants' beliefs of intelligence changeability. The scale contains eight items; four of them are reverse coded. Respondents indicated their answers on a ten-point Likert scale (from 1—“strongly disagree” to 10—“strongly agree”). This scale had good internal consistencies (α_pre_ = 0.79; α_post_ = 0.89; α_post2_ = 0.90). Higher scores on this scale indicate higher levels of intelligence changeability beliefs

#### Personality mindset scale

We used the Implicit Theory of Intelligence Scale (Dweck et al., 1995) to assess the participants' beliefs of personality changeability. The scale contains 12 items; six of them are reverse coded. Respondents indicated their answers on a ten-point Likert scale (from 1—“strongly disagree” to 10—“strongly agree”). This scale had good internal consistencies (α_pre_ = 0.81; α_post_ = 0.93; α_post2_ = 0.93). Higher scores on this scale indicate higher levels of personality changeability beliefs.

#### Academic motivation scale

We used the Academic Motivation Scale (Vallerand et al., 1992; Orosz et al., 2013) to assess the students' level of amotivation, intrinsic and extrinsic motivation toward studying. The scale contains 11 items; four items belong to the amotivation and extrinsic motivation factors and the intrinsic motivation included three items. Respondents indicated their answers on a seven-point Likert scale (from 1—“doesn't correspond at all” to 7—“corresponds exactly”). This scale had good internal consistencies (amotivation: α_pre_ = 0.86; α_post_ = 0.91; α_post2_ = 0.91; extrinsic motivation: α_pre_ = 0.78; α_post_ = 0.80; α_post2_ = 0.73; intrinsic motivation: α_pre_ = 0.82; α_post_ = 0.78; α_post2_ = 0.78).

#### Short grit scale

We used the Short Grit Scale (Duckworth and Quinn, 2009) to assess the participants' level of persistence. The scale contains eight items. Respondents indicated their answers on a five-point scale (from 1—“very much like me” to 5—“not like me at all”). This scale had good internal consistencies (α_pre_ = 0.77; α_post_ = 0.85; α_post2_ = 0.85). Higher scores on this scale indicate higher levels of persistence.

#### GPA

GPA data was available from the school's electronic diary. GPA in Hungary includes all grades including main (Mathematics, Hungarian literature and grammar, history, and foreign language) and peripheral subjects (Physics, Biology, Chemistry, Geography, Music, Informatics, PE, Arts) as well. The average GPA can be calculated based on a 1 (unsatisfactory) to 5 (excellent) grading system by aggregating and averaging the grades of the above-mentioned subjects.

### Procedure

Dissimilarly to the study of Blackwell et al. (2007) in which research was carried out by the experts, it was a train-the-trainer intervention in which one of the authors showed the training exercises to the randomly assigned teachers during 4 h and then they implemented it without further supervision. During the intervention, students were not exposed to direct persuasion regarding the content. The intervention was not framed as a helping session, but as an opportunity to learn about how one can react to difficulties.

We started the intervention at the beginning of a spring semester. The mindset intervention was held in five 45-min sessions (one per week), during a homeroom class which is designated to studies-related activities that are independent from specific classes, such as recording the attendance. In average 25 students participated in each of the classes. Homeroom teachers (teachers who have a certain class assigned to look after) of these classes were randomly assigned to experimental vs. control group. Before the first session, we assessed the students' intelligence and personality beliefs, their academic motivations, and individual differences in Grit. Table 1 provides details about the intervention protocol which borrowed some elements form the guidelines of the Heroic Imagination Project's “*The Growth Mindset: The psychology of motivation and success”* (Dickerson et al., unpublished manuscript) workshop schedule. Compared to previous interventions (Blackwell et al., 2007; Paunesku et al., 2015), in the present case, we put more emphasis on the everyday academic aspects of the incremental theory of intelligence instead of the functioning of the brain. Neuroplasticity got a larger emphasis only in the third session.

**Table 1:** **Summary of the intervention protocol**.

**Sessions**	**Intervention group—mindset**	**Control group—bystander effect**
1	Warm up, Introducing the growth mindset, exercise about a personal failure sharing it in pairs, discussions about short videos in which failures were exposed, Close up.	Warm up, Introducing the bystander effect, (1st exercise) Imagining a situation in which a person is lying on the pavement—what would you do?, (2nd exercise) Watching a short video about the bystander effect and talking about it in small groups, Close up.
2	Warm up, (1st exercise) Demonstration of a short Mindset video and based on it explanation of the difference between fixed mindset and growth mindset in terms of (a) reactions to setbacks, (b) reactions to challenges, (c) the role of effort, (d) the role of challenges, (e) reactions to criticism, (f) reactions to others' success with the active group-level activity of students. (2nd exercise) Pair then group discussion about someone who had positive expectations, personal story sharing, analysis of this behavior, its relationship with growth mindset, if you are a leader why it is important to have a growth mindset. (3rd exercise) Pair discussion about a time when the student was bad at something and now is good with the following questions: How she/he get better? How much work was needed for it? Close up, Homework.	Warm up, (1st exercise) Demonstration of a short video on lack of activity in an emergency situation and talking about the content of the video, the possible feelings and thought of the characters. (2nd exercise) Every student recorded and wrote down two own stories: one about helping someone in need and another one about not helping. Small group discussion was carried out about the stories and the feelings concerning the situations. Then these stories were anchored to the concept of pluralistic ignorance, diffusion of responsibility, and the spotlight effect which can prevent from helping in emergency situation. Close up, Homework.
3	Warm up, (1st exercise) Group level simulation of a debate in one's head to give up or work harder after a failure, gathering the good arguments to make effort despite the setback. (2nd exercise) Exposing (videos), writing down and discussing the main obstacles and writing down personal plans to overcome them. Later pair, finally group discussion. Close up, Homework.	Warm up, (1st exercise) Group level simulation of a debate in one's head to help a younger student bullied by an older one or not to help him, gathering the pro and contra arguments. (2nd exercise) Gathering the obstacles that occur in a helping situation and find the solutions to them. Close up, Homework.
4	Warm-up, (1st exercise) Writing personal plan for responding with a growth mindset every time they experience challenge or setback. Then pair, finally group discussion about these plans giving advices to each other about coping with difficulties. (2nd exercise) Writing word associations about Mindset, then gathering in group and explaining with their own words in group setting, discussing on the most interesting and surprising aspects. Close up, Homework.	Warm up, (1st exercise) Writing a personal plan for helping one of your classmates who is bullied. Then pair, finally group discussion about these plans—with further refinements in terms of planning—giving advices to each other about coping with these kind of situations. (2nd exercise) Thinking about the learnt skills and writing down the most important keywords and associations about the bystander effect. Close up, Homework.
5	Warm up, (1st exercise) Students think of someone (family member, friend) who can benefit from the mindset knowledge. First pair, then group discussion about what he would say to this person and imagination of the reactions. What is the most interesting to you regarding Mindset? (2nd exercise) Writing postcards to themselves with recommendations. Close up, Homework.	Warm up, (1st exercise) Students think of someone (family member, friend) who can benefit from the bystander effect knowledge. Write it down and some students share their “message” with the class. (2nd exercise) Summarizing the main points of helping others. (3rd exercise) Gathering what we have to take into consideration when we help others. Close up, Homework.

The control group got the same type of tasks as the intervention group, but regarding the bystander effect. The pre-test was carried out 1 week before the intervention, the post-test was 3 weeks after the 5-week long intervention and the second post-test was at the end of the school year (2 months later than the first post-test and 4 months later than the pre-test).

### Statistical analysis

To test our hypothesis that the mindset intervention would change the intelligence, personality beliefs of students, we performed 2 × 3 mixed model analyses of variance (ANOVA) with CONDITION (Growth Mindset intervention vs. control) as a between-subjects factor, and TIME (pre-intervention, post-intervention and second post-intervention) as a within-subjects factor.

First, we tested whether the growth mindset intervention changed the intelligence and personality beliefs of students by conducting pre-post-post2 comparisons on each mindset measure (intelligence and personality) among participants who participated or did not participate in the mindset intervention. Second, we tested whether the intervention changed the academic motivations and the GPA scores by conducting pre-post-post2 comparisons on the academic motivation measure (amotivation, intrinsic and extrinsic motivation) and GPA scores among participants who participated or did not participate in the mindset intervention.

Finally, we created groups based on the Grit scores (high Grit and low Grit groups) to test whether the initial extent of persistence could modify the results of the intervention. We performed 2× 2 × 3 mixed model analyses of variance (ANOVA) with CONDITON (Growth Mindset intervention vs. control) and Grit (high or low Grit scores) as between-subjects factors, and TIME (pre-intervention, post-intervention and post-intervention 2) as a within-subjects factor. We performed the same analyses as mentioned above with adding the Grit variable.

## Results

Statistical analyses were performed using SPSS 22. Means and standard deviations are provided in Table 2. The normality of the data was investigated with multiple indicators. First, the Shapiro-Wilk test indicated that the data was statistically non-normal in the case of all measurement point of amotivation (*p* < 0.001), in the case of intrinsic motivation post-test 1 (*p* = 0.033), in the case of extrinsic motivation pretest (*p* = 0.006), the second post-test (*p* = 0.042), and regarding IQ mindset post-test 1 (*p* = 0.033). Contrarily, Skewness and Kurtosis values were between ±1.5: Personality Mindset (Kurtosis_pre_ = −0.24; Kurtosis_post1_ = −0.50; Kurtosis_post2_ = −0.16; Skewness_pre_ = 0.30; Skewness_post1 =_−0.30; Skewness_post2_ = 0.36) and IQ Mindset (Kurtosis_pre_ = −0.70; Kurtosis_post1_ = −0.37; Kurtosis_post2_ = −0.46; Skewness_pre_ = −0.19; Skewness_post1_ = −0.56; Skewness_post2_ = −0.26) variables as well as for the intrinsic motivation (Kurtosis_pre_ = −0.65; Kurtosis_post1_ = −0.29; Kurtosis_post2_ = −0.55; Skewness_pre_ = −0.10; Skewness_post1_ = −0.52; Skewness_post2_ = 0.14), extrinsic motivation (Kurtosis_pre_ = −1.03; Kurtosis_post1_ = −0.42; Kurtosis_post2_ = 0.09; Skewness_pre_ = −0.34; Skewness_post1_ = −0.23; Skewness_post2_ = −0.62) and amotivation (Kurtosis_pre_ = 0.47; Kurtosis_post1_ = 1.23; Kurtosis_post2_ = −0.74; Skewness_pre_ = 1.22; Skewness_post1_ = 1.43; Skewness_post2_ = 0.82), and Grit (Kurtosis_pre_ = −0.05; Kurtosis_post1_ = −0.33; Kurtosis_post2_ = −0.51; Skewness_pre_ = −0.21; Skewness_post1_ = −0.20; Skewness_post2_ = 0.12). These Kurtosis and Skewness values are within the absolute values recommended by Curran et al. (1996) as they suggested a value of 2.0 for skewness and 7.0 for kurtosis. However, considering the results of the Shapiro-Wilk test, log10 transformation was applied for amotivation, intrinsic motivation and IQ Mindset scores.

**Table 2:** **Descriptive statistics of measures in relation to each target group**.

**Scale**	**Type of intervention**	**Mean (SD)**			**Observed range**		
		**Pre**	**Post**	**Post 2**	**Pre**	**Post**	**Post 2**
Intelligence mindset	Intervention	6.98 (1.93)	7.39 (1.78)	6.70 (2.04)	2.88–10	2.88–9.8	2–9.5
	Control	7.32 (1.63)	5.99 (2.02)	5.77 (1.69)	4.5–10	1.88–9.38	2–8.63
Personality mindset	Intervention	5.20 (1.24)	6.13 (1.62)	5.30 (2.01)	3.33–8.41	3–8.33	2.63–8.75
	Control	5.05 (1.47)	4.33 (1.50)	4.62 (1.50)	2.42–8.17	1.08–6.75	1.83–9
Amotivation	Intervention	2.32 (1.50)	1.40 (0.82)	1.98 (1.22)	1–6	1–4	1–4.50
	Control	1.97 (1.38)	2.67 (1.77)	2.42 (1.57)	1–5.75	1–6.75	1–5.50
Intrinsic motivation	Intervention	3.92 (1.76)	4.29 (1.08)	3.95 (1.29)	1–7	2–6	2–6.67
	Control	4.34 (1.38)	4.44 (1.65)	4.39 (1.49)	2–7	1.33–7	1–7
Extrinsic motivation	Intervention	4.98 (1.46)	4.93 (1.27)	4.88 (1.30)	2.5–7	2–7	1.5–7
	Control	5.48 (1.20)	5.30 (1.16)	5.28 (1.26)	2.75–7	3–7	2–7
GPA	Intervention	3.90 (.82)	–	3.87 (.82)	2.29–5.00	–	2.41–5.00
	Control	4.15 (.82)	–	4.19 (.77)	2.53–5.00	–	2.80–5.00

### Effectiveness of the mindset intervention

**Figure 1:**
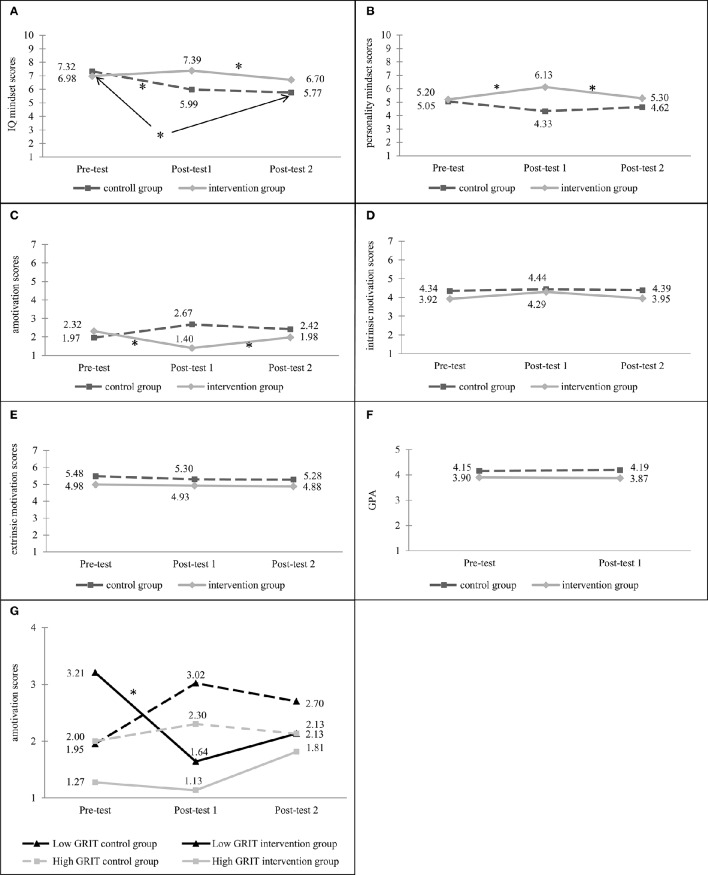
**(A)** IQ mindset scores in the intervention and control group. **(B)** Personality mindset scores in the intervention and control group. **(C)** Amotivation scores in the intervention and control group. **(D)** Intrinsic motivation scores in the intervention and control group. **(E)** Extrinsic motivation scores in the intervention and control group. **(F)** GPA scores in the intervention and control group. **(G)** Amotivation scores in High and Low GRIT intervention and control groups. ^*^p < 0.05.

The CONDITION ^*^ TIME ANOVA predicting the beliefs of the changeability of intelligence revealed significant main effects of TIME, *F*_(2, 106)_ = 4.34, *p* = 0.023, η_*p*_^2^ = 0.08, power = 0.70; however no significant main effect of CONDITION, *F*_(1, 53)_ = 2.52, *p* = 0.118, η_*p*_^2^ = 0.05, power = 0.34 was found. The interaction of CONDITION ^*^ TIME was significant, *F*_(2, 106)_ = 4.17, *p* = 0.027, η_*p*_^2^ = 0.07, power = 65. Although intelligence mindset scores did not significantly differ between the intervention and control groups at baseline (*p* = 0.381), intelligence mindset scores differed significantly in the post-test among groups (*p* = 0.015), however, the scores did not differ significantly between the second post-tests (*p* = 0.188) (see Figure 1A).

Pairwise comparisons revealed that students who participated in the intervention group did not report significantly higher intelligence mindset scores between the pre- and post-test (*p* = 0.383), while significant difference was found between the post- and the second post-test scores (*p* = 0.005), there was no significant difference between the pre- and the second post-test (*p* = 0.526). While students in the control group showed significantly lower intelligence mindset scores between pre- and post-test (*p* = 0.010) and between pre- and second post-test (*p* = 0.004), while no significant difference was found between the post- and the second post-test scores (*p* = 0.847).

The CONDITION ^*^ TIME ANOVA predicting the beliefs of the changeability of personality did not reveal significant main effects of TIME, *F*_(2, 106)_ = 0.58, *p* = 0.54, η_*p*_^2^ = 0.01, power = 0.14; however, main effect of CONDITION, *F*_(1, 53)_ = 8.11, *p* = 0.006, η_*p*_^2^ = 0.13, power = 0.80 was found. The interaction of CONDITION ^*^ TIME was significant, *F*_(2, 106)_ = 5.47, *p* = 0.006, η_*p*_^2^ = 0.09, power = 0.80. Although personality mindset scores did not significantly differ between the intervention and control groups at baseline (*p* = 0.70), personality mindset scores differed in the post-test among groups (*p* < 0.001), however in the second post-test again, there was no significant difference between the intervention and the control group (*p* = 0.164) (see Figure 1B).

Pairwise comparisons revealed that students who participated in the intervention group reported significantly higher personality mindset scores between the pre- and post-test (*p* = 0.023) and significant difference was found between the post- and the second post-test scores (*p* = 0.017), while there was no significant difference between the pre- and the second post-test (*p* = 0.80). Students in the control group showed no significant differences between pre- and post-test (*p* = 0.054), between pre- and second post-test (*p* = 0.33), and between the post- and the second post-test scores (*p* = 0.25).

### Effectiveness of the mindset intervention in academic motivations and GPA scores

In the academic amotivation scores the analysis did not reveal significant main effects of TIME, *F*_(2, 106)_ = 0.52, *p* = 0.543, η_*p*_^2^ = 0.01, power = 0.12 and CONDITION, *F*_(1, 53)_ = 2.32, *p* = 0.134, η_*p*_^2^ = 0.04, power = 0.32. The interaction of CONDITION ^*^ TIME was significant, *F*_(2, 106)_ = 6.19, *p* = 0.007, η_*p*_^2^ = 0.10, power = 80. Although academic amotivation scores did not significantly differ between the intervention and control groups at baseline (*p* = 0.341), amotivation scores differed in the post-test among groups (*p* = 0.001), however, in the second post-test again, there was no significant difference between the intervention and the control group (*p* = 0.358) (see Figure 1C).

Pairwise comparisons revealed that students who participated in the intervention group reported significantly lower amotivation scores between the pre- and post-test (*p* = 0.005) and significant difference was found between the post- and the second post-test scores (*p* = 0.006), while there was no significant difference between the pre- and the second post-test (*p* = 0.446). Students in the control group showed no significant differences between pre- and post-test (*p* = 0.089), between pre- and second post-test (*p* = 0.275), and between the post- and second post-test scores (*p* = 0.300).

In the intrinsic motivation scores the analysis did not reveal significant main effects of TIME, *F*_(2, 106)_ = 0.63, *p* = 0.494, η_*p*_^2^ = 0.01, power = 0.14 and CONDITION, *F*_(1, 53)_ = 1.19, *p* = 0.280, η_*p*_^2^ = 0.02, power = 0.19. Also, the interaction of CONDITION ^*^ TIME was not significant, *F*_(2, 106)_ = 0.95, *p* = 0.368, η_*p*_^2^ = 0.02, power = 0.19 (see Figure 1D).

In the case of extrinsic motivation, the analysis did not reveal significant main effects of TIME, *F*_(2, 106)_ = 0.27, *p* = 0.719, η_*p*_^2^ = 0.01, power = 0.09 and CONDITION, *F*_(1, 53)_ = 2.91, *p* = 0.094, η_*p*_^2^ = 0.05, power = 0.39. Also, the interaction of CONDITION ^*^ TIME was not significant, *F*_(2, 106)_ = 0.07, *p* = 0.903, η_*p*_^2^ < 0.001, power = 0.06 (see Figure 1E).

In the GPA scores the analysis did not reveal significant main effects of TIME, *F*(_1, 72)_ = 0.07, *p* = 0.79, η_*p*_^2^ = 0.00, power = 0.06 and CONDITION, *F*_(1, 72)_ = 2.35, *p* = 0.13, η_*p*_^2^ = 0.03, power = 0.33. Also, the interaction of CONDITION ^*^ TIME was not significant, *F*_(1, 72)_ = 2.60, *p* = 0.11, η_*p*_^2^ = 0.04, power = 0.36 (see Figure 1F).

### Effectiveness of the mindset intervention in low and high GRIT score groups

We examined whether participants' level of persistence (high/low GRIT) would moderate the effectiveness of mindset interventions. Based on GRIT scores measuring the extent of persistence (*M* = 28.15; *SD* = 5.30), the sample was split into two groups (median split) to distinguish between participants who had lower level of persistence (*M* < 28) and those who had higher level of persistence (*M* ≥ 28). We then conducted a 2 (CONDITION) ^*^ 3 (TIME) ^*^ 2 (GRIT: High/Low) ANOVA to predict the changes in mindset beliefs, in academic motivation and in GPA. There were 14 participants in the low GRIT intervention group, 12 in the high GRIT intervention group, 14 in the low GRIT control group and 15 in the high GRIT control group.

In the case of academic amotivation, beyond the effects for CONDITION and TIME reported above, this analysis revealed significant main effect for GRIT, *F*_(1, 51)_ = 7.81, *p* = 0.007, η_*p*_^2^ = 0.13, power = 0.78 (see Figure 1G). GRIT did not significantly interact with either TIME, *F*_(2, 102)_ = 1.24, *p* = 0.27, η_*p*_^2^ = 0.02, power = 0.19 or CONDITION, *F*_(1, 51)_ = 1.52, *p* = 0.22, η_*p*_^2^ = 0.03, power = 0.23. However, the three-way interaction between CONDITION, TIME, and GRIT was significant, *F*_(2, 102)_ = 3.51, *p* = 0.048, η_*p*_^2^ = 0.06, power = 0.56.

Pairwise comparisons revealed that students who participated in the intervention group with lower persistence (low GRIT group) reported significantly lower amotivation scores between the pre- and post-test (*p* = 0.009), however, no significant difference was found between the post- and the second post-test scores (*p* = 0.051) and between the pre- and the second post-test (*p* = 0.102). Pairwise comparisons also revealed that students who participated in the intervention group with higher persistence (high GRIT group) reported no significantly lower amotivation scores between the pre- and post-test (*p* = 0.075), between the post- and the second post-test scores (*p* = 0.065), and between the pre- and the second post-test (*p* = 0.185). Moreover, pairwise comparisons revealed that students who participated in the control group with lower persistence (low GRIT group) did not report significantly higher amotivation scores between the pre- and post-test (*p* = 0.096), whereas the difference was not significant between the post- and the second post-test scores (*p* = 0.311), and between the pre- and the second post-test (*p* = 0.297). Pairwise comparisons revealed that students who participated in the control group with higher persistence (high GRIT group) reported no significantly higher amotivation scores between the pre- and post-test (*p* = 0.568), between the post- and the second post-test scores (*p* = 0.770), and between the pre- and the second post-test (*p* = 0.688). In the case of IQ mindset, personality mindset, intrinsic motivation, extrinsic motivation, and GPA-change, no effects of GRIT can be observed.

## Discussion

Compared to previous successful Growth Mindset interventions (Aronson et al., 2002; Good et al., 2003; Blackwell et al., 2007; Paunesku et al., 2015; Yeager et al., 2016), the present one did not achieve its goals in terms of belief-, motivation- and GPA-change. Regarding the short-term positive consequences, the present form of growth mindset intervention resulted in more incremental personality beliefs, and it sustained the initial level of the amotivation and IQ mindset scores. However, in the control group, the personality mindset scores stayed at the initial level, and students in this condition also reported more fixed intelligence mindset scores and higher level of amotivation. Therefore, the short-term effect of this growth mindset intervention kept IQ mindset high and amotivation low, while at the same time inducing more incremental personality beliefs. However, all of these effects disappeared by the end of the semester. Furthermore—according to a further analysis focusing the role of Grit as a more stable personality characteristic—we found that the short term drop of amotivation can be attributed to those students who are less persistent. Among the low Grit students who participated in the growth mindset treatment, we measured a short-term drop of amotivation, but this effect also disappeared by the end of the semester. Finally, the growth mindset intervention could not improve the GPA of the students. In sum, we found some short-term belief-related and motivational effects, but no long-term effects were sustained.

Some previous studies did not support the beneficial effect of the growth mindset interventions on motivation, academic performance beliefs, study skills, and math performance (Dommett et al., 2013). However, at least Dommett et al.'s (2013) study could demonstrate a small but significant long-term effect with the increased incremental intelligence beliefs. Donohoe et al.'s (2012) used a similar growth mindset intervention in an online setting and they had similarly short-term positive results in terms of resiliency and sense of mastery that disappeared after 3 months. Dommett et al.'s (2013) results, along with the present ones, show that mindset beliefs—in the field of personality and intelligence—are temporal malleable in given circumstances and they can return to their initial state in the long run.

One of the most interesting results is related to the question as to why growth mindset beliefs of the control group participants became more fixed over time. One of the potential explanation is related to the fluid aspect of the school context (Spitzer and Aronson, 2015). It is possible that when the end of semester approached, students experienced several situations—criticism, failures, setbacks—which made them doubt their abilities. Furthermore, it is also possible that their grades and the feedbacks of their teachers did not change in the expected way or to the expected extent which in turn had a negative effect on their growth mindset beliefs.

As researchers with less experience in social psychological interventions, first, we interpret these results in the light of the guidelines of more experienced experts of this field. Second, we aim to share our experience with this specific intervention, hoping that it might be valuable for similarly less experienced researchers.

Garcia and Cohen (2011) used the interpretation of Lewin (1948, 1951) concerning the classroom which is a relatively stable tension system including dynamic forces of interaction. Social psychological interventions can modify this tension system in a successful way if they take into consideration core elements identified by previous research (Garcia and Cohen, 2011; Yeager and Walton, 2011; Walton, 2014). These seven core elements—psychological precision, targeting specific groups, appropriate timing, creating recursive processes, embedding in the appropriate context, avoiding persuasive appeals and framing as learning opportunity—are depicted in Table 3 in the light of the present study design. The present results suggest that an intervention can be a holistic context in which changing one parameter can lead to different results.

**Table 3:** **Comparing the requirements of social psychological interventions (Garcia and Cohen, 2011; Yeager and Walton, 2011; Walton, 2014) with the elements of the present study**.

**Requirements of a good social psychological intervention**	**How it appeared in the present study**
1. Psychologically precise (theory and tools)	It used the Mindset theory and valid measurements
2. Targets a specific group	High achievers were targeted
3. Appropriate timing	Second semester of second year in high school
4. Recursive processes	Weekly, 1-h session for 5 weeks
5. Appropriate context	Classroom context in which the form master conducted the intervention and each student participated in the sessions
6. Not using direct persuasive appeal	Students shared their experiences with each other, not direct, one-way lecture was given about the subject
7. Not help, but give an opportunity	The training was framed as a learning opportunity

### Psychological precision

The present intervention was based on the Mindset theory of Dweck et al. (1995). Although this training focused on the analogy of the plasticity of the brain to some extent, it was not as emphasized as in the case of both previous online (Paunesku et al., 2015) and offline Mindset interventions (Blackwell et al., 2007). We rather focused on more applied aspects of growth mindset, such as what students can do with potential obstacles in difficult situations (see Table 1, second session) as well as we demonstrated the internal dialog between growth and fixed mindset in order to provide practical guidelines for using growth mindset in different ways in diverse situations (see Table 1, third session). The lack of efficacy of the intervention can be attributed to the lack of such analogy which can be easily objectified by the students. Besides this alternation, the intervention was based on solid theoretical background and intended to change only one specific belief. In sum, besides the solid theoretical background, recurring, very illustrative analogies—such as the brain-muscle one in the original Growth Mindset interventions—can also contribute to the effectiveness.

### Targeting specific groups

In most of the Mindset interventions, specific target groups were low achievers (Blackwell et al., 2007; Paunesku et al., 2015), low income students (Good et al., 2003) or minority students (Aronson et al., 2002). In these studies, the effect of the intervention was visibly more salient than among other experimental group members. In the present case, students with relatively good grades with no minority background were in the focus of investigation. Among these students, the training was effective in short-term regarding their amotivation, whereas this improvement diminished by the end of the semester.

In the present case, neither extrinsic nor intrinsic motivation changed in long-term, similarly to GPA. Very probably, in the case of these students, measuring challenge-taking might be more beneficial. These students were possibly less exposed to failures than students with worse grades. However, in this explanation, we cannot ignore the importance of subjective experience of failure. Further investigations are needed to support that students with good grade are less exposed to failures. It is possible to expect that students with good grades in the Hungarian educational context have comparable results to underprivileged students in the US. However, our results did not support this assumption.

### Appropriate timing

The third point refers to the appropriate timing. In the present case, it was not at the beginning of the high school (9th grade), but at the beginning of the second semester of the 10th grade. According to both Yeager and Walton (2011) and Garcia and Cohen (2011), interventions in the transition points are more effective. We assume that the stressful nature of the transition context is important because it can allow context-dependent learning. If a well-targeted intervention (beliefs in change through effort) can help the student with immediate positive feedbacks in these transitional circumstances (i.e., positive feedbacks such as less failure in longer term, better grades attributed to more effort or better strategy, etc.), then the recently learnt strategies can be reinforced and more probably used in further stressful situations. In the present case, coping routines related to high school context had already stabilized. Supposedly, changing these already established attributions might need more in-depth and lengthier interventions. In sum, in the present case, the timing was sub-optimal. Future interventions should focus more on the appropriate timing.

### Creating recursive processes

The fourth element refers to recursive processes. Both one session (Paunesku et al., 2015), two-session (Yeager et al., 2016) and multiple session (Blackwell et al., 2007) interventions can be effective. The recursive nature of the intervention can appear through the number of direct interventions and the processes catalyzed by previous session. Obviously, it is more economic if one session can catalyze further long-term recursive processes which leads to far-reaching positive consequences. In the present case, compared to Blackwell et al.'s (2007) eight sessions, we had fewer sessions (five) with less results. There are many reasons why it might have been less effective.

Paradoxically, longer interventions do not help in reinforcing the recursive processes, because they can provide “too many” information, allowing room for diverse interpretations. For example, in the present case, thanks to the numerous activities, some students might put more emphasis on the role of efforts, while others might put more emphasis on choosing the appropriate problem-solving strategies. Therefore, as a consequence of this biased representation of Growth Mindset, their effort beliefs can become rather different. This belief bias might influence further catalyzing processes, because resolving a problem attributed to efforts vs. careful strategy choice can reinforce more efforts and less experimenting with newer strategies. The more components of the Growth Mindset are in the focus of a longer intervention, the more alternative (mis)interpretation and bias can be expected. According to Dweck (2016), such misinterpretations can lead to a false growth mindset, including elements such as effort is more important than asking help from others or trying out new strategies. In order to identify the specific effect of the intervention, differentiated measurement of these belief-facets (i.e., efforts, strategies, asking help from others) might be beneficial in future studies. Possibly, physical tools—such as posters in the classrooms depicting infographics on the appropriate content of the Growth Mindset—could be an adequate reminder for this purpose. In sum, in the case of social psychological interventions, less can be more.

### Embedding in the appropriate context

The fifth element refers to the appropriate context. In the present case, the teachers started to implement the mindset module exercises in their school context. The training neither aimed at changing the school climate, nor directly changing the teacher's teaching practices; it only provided knowledge about Mindset theory through the above mentioned exercises. Naturally, we did not intend to deeply change the school context in itself. Teachers—who volunteered for participating in this study and who were randomly assigned to groups—could have teaching practices which reflect on fixed mindset and growth mindset as well. Unfortunately, teachers' initial mindset beliefs were not assessed in the present study. However, these initial beliefs can be critical in terms of teaching practices and how they conduct the mindset intervention in the classroom.

Another important issue is that we supposed that a 4-h long training can change the teachers' intelligence beliefs toward the growth mindset which can be transmitted through the exercises described above. Many factors can influence this context: (a) students' attitudes toward the teacher who led the training, (b) how much growth mindset appears the given teacher's educational practices which can validate the message of the training, (c) the attitude of the other teachers at the school concerning the notions of the growth mindset, (d) the presence or lack of standardized testing in terms of grading, (e) peer norms concerning Growth Mindset, etc. As far as we know, only a few studies examined these factors in details in the light of the effectiveness of the interventions. Future studies are needed in order to identify the relative importance of these (and other) contextual factors.

### Avoiding persuasive appeals and framing as learning opportunity

Concerning the last two characteristics of wise social psychological interventions—the avoidance of using persuasive appeals and not framing the intervention as helping but as an opportunity—the present Mindset training met these requirements (Yeager and Walton, 2011; Walton, 2014). The exercises summarized in Table 1 are neither directly persuasive, nor framed as helping.

One might think that a student (or an adult) can hold growth mindset irrespectively to the environmental context or feedback. Despite the weaknesses of the present study, it became evident that Mindset beliefs can temporarily change in a beneficial direction, but in the long run, they can change back to their pre-intervention level. Further research is needed to explore the interactions between the relevant social situations and the temporal changes on the continuum of fixed and growth mindset beliefs. However, it appears that the present train-the-trainer intervention did not lead to a long term positive mindset change. In sum, Mindset beliefs are malleable and not only in the good direction. Future research is needed to explore which contextual variables can change back an intervention-induced growth mindset to the baseline.

### The role of grit in growth mindset interventions among students with high GPA

Despite the similar theoretical roots of mindset (Hong et al., 1999) and Grit (Duckworth et al., 2007) theories in terms of persistence and effort, as far as we know, no previous research was conducted to measure the link between them in an intervention study. Despite low Grit students' intrinsic, extrinsic motivations and grades did not differ, their amotivation decreased temporarily more than amotivation of high Grit students. Therefore, we assume that high-Grit good-grader students' amotivation basically stayed on a stable low level. On the other hand, those good-graders who were low on Grit measure felt less that they were wasting their time at school after 3 weeks of the intervention than before. This effect was quite large: the averages of the amotivation scores dropped by almost 40% by the first post-test. If this level of amotivation could be stabilized among low Grit good-graders, then these students could benefit a lot from such interventions. Unfortunately, according to the results, this effect also disappeared by the end of the semester. Furthermore, it is important to note that the pre-test amotivation scores of the high-grit and low-grit intervention group were different which may also bias the results.

### Limitations and future directions

One of the merits of the present study that it opens several questions; however, besides the above-mentioned principle-based issues, we can mention more specific methodological mistakes and limitations. These can also be instructive to those—similarly to the first four authors of the present study—who start to implement similar social psychological interventions in educational context. First, we found significant changes only in the case of self-reporting; further studies should use similar behavioral measures as the Yeager et al. (2014, 2016) studies used. Furthermore, it would be important to measure the perceived change reported by teachers, parents and peers (as norms how the class can accept failures). Second, high drop-out rate could be avoided by online data gathering (instead of paper and pencil solutions) and using the participants' name instead of code-names. Third, it might be a fruitful way to give more in-depth trainings to the teachers instead of 4-h trainings and measure how much their teaching practices changed. We might think that their beliefs changed, but this could not manifest in their instructional behavior. Supposedly, teachers who provide such knowledge via trainings should be trained to the implementation of these beliefs in her/his instructional practices. Furthermore, they might be trained to recognize the signs (of communication or behavior) when they teach with fixed mindset. Fourth, future interventions should choose randomization of students instead of teachers or classrooms, similarly to Paunesku et al. (2015). Fifth, we assume that a good intervention can be implemented not only in the case of students, but to the parents and teachers as well. This multidirectional intervention could create a supportive social context in which growth mindset can be catalyzed in a more efficient way. Sixth, the sample size hand in hand with the power were low, therefore it is important to have a larger initial sample size and in the present case be cautious regarding even with the short term results.

## Conclusion

The goal of the present study was to assess the effectiveness of a train-the-trainer mindset intervention among Hungarian 10th grade students with high GPA. Teachers led the intervention after a 4-h long training which highlighted the most important aspects of Hong et al. (1999) mindset theory. The results suggest that growth beliefs of personality changed in a positive way (i.e., students had more growth mindset) 3 weeks after the intervention. Growth mindset IQ scores were sustained as a result of the intervention, whereas we found a significant drop of this measure in the control group after 3 weeks. Furthermore, the amotivation score of the intervention group reduced compared to both the baseline measure and the control group. However, no intrinsic and extrinsic motivational change was measured. Students with good grades and low Grit scores reported lower amotivation compared to grittier high achievers in the first post test. However, by the end of the semester, these positive changes disappeared. Furthermore, their GPA did not improve as a result of the intervention. These results show that mindset beliefs can change back to their pre-intervention state. Several possible explanations exist as to why this intervention was not successful in the long run; therefore, future research is needed to explore the boundaries of the effectiveness of growth mindset interventions.

## Author contributions

GO designed the study, analyzed the data, interpreted the data and drafted the manuscript. SP designed the study, collected data, and drafted the manuscript. BB analyzed the data, drafted the manuscript and interpreted the data. IT analyzed the data, drafted the manuscript and interpreted the data. RB designed the study, interpreted the data and drafted the manuscript. All authors commented on the draft and contributed to the final version, approved the publication of the manuscript, and agreed to be accountable for all aspects of the work.

### Conflict of interest statement

The authors declare that the research was conducted in the absence of any commercial or financial relationships that could be construed as a potential conflict of interest.
